# A Zinc Metalloprotease *nas-33* Is Required for Molting and Survival in Parasitic Nematode *Haemonchus contortus*

**DOI:** 10.3389/fcell.2021.695003

**Published:** 2021-07-13

**Authors:** Yan Huang, Jie Wu, Xueqiu Chen, Danni Tong, Jingru Zhou, Fei Wu, Hui Zhang, Yi Yang, Guangxu Ma, Aifang Du

**Affiliations:** College of Animal Sciences, Zhejiang Provincial Key Laboratory of Preventive Veterinary Medicine, Institute of Preventive Veterinary Medicine, Zhejiang University, Hangzhou, China

**Keywords:** nematode astacin, *nas-33*, *gpb-1*, molting, anthelmintic target

## Abstract

Molting is of great importance for the survival and development of nematodes. Nematode astacins (NAS), a large family of zinc metalloproteases, have been proposed as novel anthelmintic targets due to their multiple roles in biological processes of parasitic nematodes. In this study, we report a well conserved *nas-33* gene in nematodes of clade V and elucidate how this gene is involved in the molting process of the free-living nematode *Caenorhabditis elegans* and the parasitic nematode *Haemonchus contortus*. A predominant transcription of *nas-33* is detected in the larval stages of these worms, particularly in the molting process. Knockdown of this gene results in marked molecular changes of genes involved in cuticle synthesis and ecdysis, compromised shedding of the old cuticle, and reduced worm viability in *H. contortus*. The crucial role of *nas-33* in molting is closely associated with a G protein beta subunit (GPB-1). Suppression of both *nas-33* and *gpb-1* blocks shedding of the old cuticle, compromises the connection between the cuticle and hypodermis, and leads to an increased number of sick and dead worms, indicating essentiality of this module in nematode development and survival. These findings reveal the functional role of *nas-33* in nematode molting process and identify astacins as novel anthelmintic targets for parasitic nematodes of socioeconomic significance.

## Introduction

Both free-living and parasitic nematodes develop through four to five larval stages, which are distinguishable by their different size and separated by the temporal shedding of cuticle (i.e., molting). The cuticle is a crucial structure that maintains post-embryonic body shape, acts as an exoskeleton, and permits mobility and elasticity of nematodes (Bird and Bird, 1991; Page and Johnstone, 2007), whereas molting is a series of biological processes including separation of the surface coat from the epidermis (i.e., apolysis), synthesis of a new cuticle during an inactive stage (i.e., lethargus), and shedding of the old cuticle (i.e., ecdysis) (Page, 2001). Proper synthesis of cuticle and regular molting between two life stages are essential for the survival and development of nematodes in the environment or within host animals. In particular, the cuticle of parasitic nematodes is the interface of host-parasite interactions, playing roles in immune recognition and immune evasion within host animals (Maizels, 2013). A wealth of information about the cuticle and the molting process is now available for nematodes, predominantly based on the nematode model organism *Caenorhabditis elegans* (Politz and Philipp, 1992).

Molecules involved in molting include zinc metalloproteases, leucine amino-peptidases and cysteine proteases (Rogers, 1982; Gamble et al., 1989; Lustigman, 1993). Astacins are a large family of zinc metalloproteases belonging to the M12A family, which was first reported in the crayfish *Astacus astacus* (Pfleiderer et al., 1967; Mohrlen et al., 2006). In *C. elegans*, there are 40 genes coding for nematode astacins (*nas*), representing six subgroups (I, II, III, IV, V, and VI) of astacin-like proteins based on the deduced domain architectures (Mohrlen et al., 2003). These molecules have been reported predominantly expressed in the pharynx, intestine, body wall muscle and hypodermis, with a few of them expressed in neurons and the reproductive tissues of *C. elegans* (Park et al., 2010). Members of the astacin family exhibit numerous physiological functions in hatching, digestion, peptide processing and pattern formation. In particular, *dpy-31* (also known as *nas-35*), *nas-36* and *nas-37* have been reported to be involved in the nematode molting process (Maeda et al., 2001; Kamath et al., 2003). Specifically, worms lacking *dpy-31/nas-35* showed a dumpy appearance (Novelli et al., 2004, 2006), whereas suppression of *nas-36* and *nas-37* led to molting defect and temperature-sensitive lethal phenotype (Davis et al., 2004; Suzuki et al., 2004). By contrast, only a few astacin-coding genes have been identified in parasites, such as *Brugia malayi* (a filarial worm of medical importance), *Haemonchus contortus* and *Teladorsagia circumcincta* (highly pathogenic worms of veterinary significance) (Stepek et al., 2010, 2011), and little is known about their functional details in parasitic nematodes. Nonetheless, chemical inhibition of DPY-31/NAS-35 in these parasitic nematodes elicited severe dumpy and immobile phenotypes (France et al., 2015; Stepek et al., 2015), suggesting the possibilities of NAS as drug targets in major parasitic worms of socioeconomic importance. Protease inhibitors that can specifically bind to certain proteases have been used in the therapeutic treatment of parasitic diseases (Shamsi et al., 2016; Deu, 2017). For instance, vinyl sulfone cysteine protease inhibitor K11777 (a substrate-based inhibitor of the gut-associated cathepsin B1 cysteine protease) showed significant efficacy on schistosomiasis in murine model (Abdulla et al., 2007). In addition, screening of inhibitory compounds targeting essential proteases has been an emerging area for *Plasmodium falciparum* (Sharma et al., 2015; Roy, 2017; Singh et al., 2021). Therefore, a better understanding of the functional roles of *nas* genes in free-living and parasitic nematodes should underpin the biological understanding of this metalloprotease-coding gene and lay a basis for the discovery of novel interventions.

In this study, we report a zinc metalloprotease coding gene *nas-33* that is well conserved in clade V parasitic nematodes, and elucidate the essential roles of this gene in molting process in the free-living nematode *C. elegans* and the parasitic nematode *H. contortus*. Novel insights into the essentiality of *nas-33*, which represents a conserved nematode-specific gene family, should lay a solid foundation for the discovery and development of novel anthelmintics.

## Materials and Methods

### Nematodes

*Caenorhabditis elegans* N_2_ strain was acquired from the Caenorhabditis Genetics Center (CGC), maintained on nematode growth media (NGM) plates at 20°C following the standard protocol (Brenner, 1974). Gravid worms were bleached with hypochlorite solution to collect eggs, which were then incubated in M9 buffer on a rotator for 24 h at 20°C to synchronize all animals at the first larval (L1) stage (Sulston and Hodgkin, 1988). By contrast, *H. contortus* (ZJ strain; anthelmintic susceptible) were maintained in Hu sheep under a helminth-free condition as described previously (Yan et al., 2014). Adult worms were collected from the abomasa of infected sheep, and eggs were isolated from the uteri of adult female worms, placed on 2% agar plates and cultured at 28°C for 7 days to synchronize all worms at the third larval (L3) stage.

### Molecular Cloning and Sequence Analysis

Genomic DNA and total RNA were extracted from the adult worms of *H. contortus* using a TIANamp Genomic DNA kit (Tiangen Biotech Co., Ltd., Beijing) and the Trizol reagent (Invitrogen, United States), respectively. The first strand cDNA was synthesized using a First Strand cDNA Synthesis Kit (Toyobo Co., Ltd., Japan). Rapid amplification of cDNA ends (RACE) was conducted using the 5′- and 3′-Full RACE kit (Takara Biotechnology Co., Ltd.) to extend a sequence fragment HCISE01811400.t1 in the Sanger database^1^, a potential *nas-33* homolog in *H. contortus*. PCR products were cloned into a pMD19-T vector and sequenced. Based on the obtained sequence, primers were designed to perform a Genome Walking experiment to acquire the flanking sequences. Primers used were listed in Supplementary Table 1. Functional domain predictions were carried out by searching the predicted amino acid sequences against NCBI^2^ and InterPro^3^ databases. Sequence alignment and phylogenetic analyses were performed using MEGA5.

### Quantitative Real-Time PCR (qRT-PCR)

Arrested L1s of *C. elegans* were placed on NGM plates seeded with *Escherichia coli* strain OP50 and cultured at 20°C. Nematode samples were collected every 2 h until 40 h post incubation for RNA extraction. Transcriptional alteration of *Ce-nas-33* during the development was determined by qRT-PCR using SYBR^®^ Green PCR Master Mix (Toyobo, Japan) on a T100 Real-Time PCR System (Bio-Rad, United States). Actin coding gene *act-1* was used as an internal control, and cathepsin L-like cysteine protease coding gene *cpl-1, nas-37* (apolysis), collagen coding gene *col-12* (late lethargus), thioredoxin reductase coding gene *trxr-1* or glutathionine reductase coding gene *gsr-1* (ecdysis) were selected as markers for molting processes (Hashmi et al., 2002; Davis et al., 2004; Stenvall et al., 2011). Differently, synchronized L3s of *H. contortus* were used to orally infect sheep, and to collect ∼10,000 eggs, ∼8,000 L1s, 8,000 L2s, 6,000 L3s, 100 L4s and adults for RNA extraction. For each sample, 0.5-1 μg of total RNA was used to prepared cDNA for qRT-PCR performed to determine the transcriptional levels of *Hc-nas-33* in different developmental stages of *H. contortus*. In particular, transcription of *Hc-nas-33* in larvae during L1, L1-L2 molting, L2 and L2-L3 molting were measured. 18S rRNA was used as an internal control in *H. contortus*. Primer sets used can be found in Supplementary Table 1. All experiments were conducted at least three times, and the qRT-PCR data were analyzed using the 2^–△*Ct*^ method.

### RNA Interference (RNAi)

A feeding method was employed to conduct the RNAi assay on *H. contortus* (Zawadzki et al., 2012). Specific PCR primers were designed to amplify *Hc-nas-33* (613–1,569 nt). The PCR products were cloned into the L4440 vector, then transformed into *E. coli* HT115 strain (DE3) cells. Beta-tubulin isotype-1 coding gene *Hc-iso-1* was used as a positive control in *H. contortus* RNAi assays (Samarasinghe et al., 2011), whereas *Bt-cry1Ac* from *Bacillus thuringiensis* (GenBank Accession No. GU322939.1) was used as an “irrelevant” control. Primers used were listed in Supplementary Table 1. Eggs (*n* ≈ 4,000) of *H. contortus* were sterilized with antibiotic-antimycotic, washed thoroughly, and incubated with the transformed bacteria at 28°C for 6–10 days. Hatching rate and subsequent larval development of *H. contortus* were monitored under a microscope on days 1, 3, and 7. On day 3, ∼4,000 larvae were harvested for the extraction of RNA and synthesis of cDNA. Gene knockdown of *Hc-nas-33* and associated transcriptional alterations of genes involved in cuticle synthesis (*col-12*, *col-14*, cuticle procollagen coding gene *dpy-5* and *dpy-13*), ecdysis (serine/threonine protein kinase coding gene *nekl-2*, ankyrin repeat and sterile alpha motif domain containing protein coding gene *mlt-3* and tropomyosin coding gene *lev-11*) and remodeling of cuticle-epidermis linkage (muscle attachment abnormal associated gene *mua-3* and myotactin coding gene *let-805*) in RNAi-treated worms were assessed by qRT-PCR.

### Yeast Two-Hybrid Screening

Total RNA was isolated from the L3s of *H. contortus* to construct a cDNA library, which was then transformed into the Y187 yeast strain. The full-length *nas-33* cDNA was amplified and subcloned into pGBKT7, then transformed into yeast strain Y2H Gold. The NAS33 protein was used as a bait to screen the yeast cDNA library according to the manufacturer’s user guide (Matchmaker^®^ Gold Yeast Two-Hybrid System User Manual). Clones grown on the SD/-Leu/-Trp/-His/-Ade plates were confirmed by further selection and used to extract plasmids for sequencing inserts. Among the candidate genes after library screening, one insert was predicted to encode a 321 amino acid polypeptide that shares homology with the guanine nucleotide-binding protein subunit beta-1, which was renamed here as *Hc-gpb-1*.

### *In vitro* Pull-Down Assay

Bait protein (GST-fused *Hc*-NAS-33) was expressed using a Bac-to-Bac Baculovirus Expression System, immobilized with GST beads at 4°C for 4 h and then washed by phosphate buffer saline (PBS) containing 1% Triton X-100. Potential prey protein (HA-tagged *Hc*-GPB-1) was produced in HEK 293T cells transfected with pGEX-4t-1-*Hc-gpb-1*. Immobilized bait protein was incubated with 300 μl cell lysates containing HA-tagged protein at 4°C for 2 h, followed by washing with PBS. Protein-protein interaction complex was eluted, resuspended in 40 μl 2 × SDS loading buffer, boiled and separated by SDS-PAGE. Anti-GST and Anti-HA antibodies were used to analyze the interaction of *Hc*-NAS-33 and *Hc*-GPB-1.

### Co-immunoprecipitation (Co-IP)

HA-tagged *Hc*-NAS-33 and FLAG-tagged *Hc*-GPB-1 proteins were prepared from transfected HEK 293T cells, then incubated with anti-FLAG agarose at 4°C for 2 h. After that, the proteins were washed with immunoprecipitation buffer (136.89 mM NaCl, 2.67 mM KCl, 8.1 mM Na2HPO4, 1.76 mM KH2PO4 and 0.5% Tween 20) for eight times, mixed with 50 μl 5 × SDS loading buffer and boiled for 10 min. Protein samples were separated by SDS-PAGE, and subjected to Western Blot analysis. Anti-HA and anti-FLAG antibodies were used to detect fused proteins *Hc*-NAS-33 and *Hc*-GPB-1, respectively. Co-IP of *Hc*-NAS-33 and *Hc*-GPB-1 with exchanged tags was also performed.

### Co-localization

Promoters of *Ce-nas-33* (sequence between K04E7.4 and *Ce-nas-33* start codon) and *Ce-gpb-1* (sequence between F44E5.14 and *Ce-gpb-1* start codon), as well as sequences 2,000 nt upstream *Hc-nas-33* and *Hc-gpb-1* were used to drive gene expression of *Hc-nas-33* and *Hc-gpb-1* in *C. elegans*, respectively. Plasmid expressing *Hc*-NAS-33-GFP (pPD95_77-*Ce*p*-Hc-nas33*) was constructed by inserting the promoter of *Ce-nas-33* cloned from *C. elegans* DNA and *Hc-nas-33* full-length cDNA into the germline expression vector pPD95_77 via *Bam*H I and *Kpn* I restriction site successively. As the same, the promoter of *Ce-gpb-1* was cloned into pPD95_77 at *Bam*H I restriction site and *Hc-gpb-1*-mCherry overlapped sequence was inserted afterward to obtain plasmid expressing *Hc*-GPB-1-mCherry (pPD95_77-*Ce*p*-Hc-gpb1*-mCherry). Recombinant plasmids were microinjected into the gonads of young adult worms as described previously (Mello et al., 1991), together with pRF4 plasmids (50 ng/μl) introducing mutant allele of *rol-6* gene. F2 larvae with a roller phenotype were selected to examine the expression patterns of fusion proteins in the transgenic worms using a fluorescent microscope (Zeiss LSM 780). In addition, *Hc-nas-33* and *Hc-gpb-1* was cloned into C_2_-EGFP vector and pcDNA3 (+)-mCherry vector, respectively. Recombinant plasmids were co-transfected into the human embryonic kidney 293T (HEK 293T) cell line using Lipofectamine 2000 (Invitrogen). Transfected cells were further cultured at 37°C in 5% CO_2_ for 24–48 h, then stained with DAPI for 30 min at room temperature. GFP and mCherry expression in HEK 293T cells were analyzed using a fluorescent microscope (Zeiss LSM 780).

### Transmission Electron Microscopy (TEM)

About 2000 RNAi-treated and -untreated worms were collected on day 5, washed in physiological saline and fixed in 2.5% glutaraldehyde and 1% Triton X-100 and 0.1 M sodium phosphate buffer at 4°C for 7 days. Fixed samples were mounted into agar blocks and postfixed in 1% OsO4 and 0.1 M sodium phosphate buffer for 2 h, and further processed for dehydration and infiltration. Processed specimens were placed in Eppendorf tubes containing Spurr resin and heated at 70°C overnight, then sectioned and stained with uranyl acetate for 5 min and alkaline for 10 min. TEM scanning was performed using a Hitachi Model H-7650 TEM microscope. Micrographs (*n* = 4) captured at the middle of worms were selected for analysis. Thickness of the worm cuticle was measured using the ImageJ.

### Developmental and Survival Assay

Separate (*Hc-nas-33* or *Hc-gpb-1*) and simultaneous (*Hc-nas-33* + *Hc-gpb-1*) RNAi assays were conducted on the early larval stage of *H. contortus* using the feeding method described above. Gene knockdown analyses were performed to estimate the transcriptional association of these two genes in the treated worms, using qRT-PCR as described above. Primer sets used were listed in Supplementary Table 1. Developmental and survival variations of treated worms were assessed in terms of the morphology, molting and morbidity (i.e., sickness and death) of free-living L1s, L2s, and L3s (infective) of *H. contortus*. In brief, three sub-samples of 200 μl culture medium (containing about 200 larvae) were taken from the culturing system and transferred into 6-well culture plate on days 3 and 7. The treated larvae were examined by microscopy, in aspects of larval development and survival. Experiments were repeated for three times on different days.

### Statistical Analysis

Data were presented as mean ± standard deviation (SD). Statistical analyses of gene transcription, cuticle thickness and larval development were carried out by Student’s *t*-test with the GraphPad Prism 8 (GraphPad Software, United States). *P* < 0.05 was considered as statistically significant difference.

## Results

### *nas-33* Is Relatively Conserved in Free-Living and Parasitic Nematodes

**FIGURE 1:**
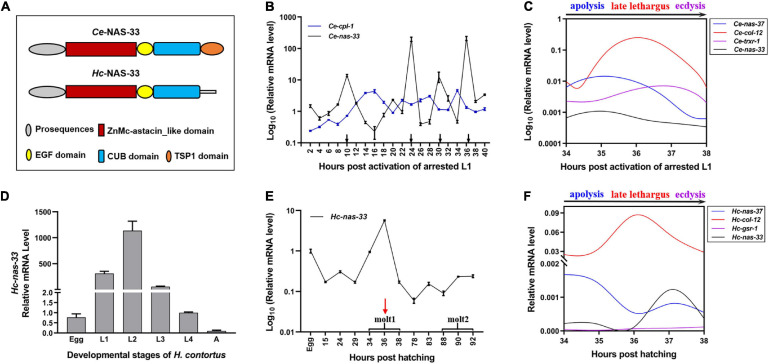
Developmental transcription analysis of *nas-33* in *Caenorhabditis elegans* and *Haemonchus contortus*. **(A)** Schematic diagram for domain architectures of *Ce*-NAS-33 and *Hc*-NAS-33. **(B)** *Ce-nas-33* shows a tightly regulated transcriptional pattern across four molting periods (black arrows) of *C. elegans*. **(C)** Transcriptional alterations of marker genes *nas-37*, *col-12*, *trxr-1* for apolysis, late lethargus and ecdysis, and *nas-33* during the molting of *C. elegans*. **(D)** Transcription levels of *Hc-nas-33* in different development stages of *H. contortus*. **(E)** *Hc-nas-33* shows a transcriptional peak during the first molting of *H. contortus*. **(F)** Transcriptional alterations of marker genes *nas-37*, *col-12*, *gsr-1* for apolysis, late lethargus and ecdysis, and *nas-33* during the first molting of *H. contortus*. Error bars indicate mean ± standard deviation (SD). A Non-linear Curve Fit is performed to indicate the dynamic transcriptional changes of marker genes involved in the molting processes.

Apart from *C. elegans*, orthologs of *nas-33* were commonly identified in nematodes of clade V, including the free-living *Pristionchus pacificus* and the parasitic *Ancylostoma caninum*, *A. ceylanicum*, and *H. contortus*. Specifically, the cDNA-confirmed *Hc-nas-33* was 1714 bp (GenBank accession No. MT891116) with a 66-bp 5′ UTR and a 79-bp 3′ UTR (Supplementary Figure 1), which encoded a protein containing a ZnMc-astacin_like domain, an EGF domain and a CUB domain (Figure 1A). Notably, there was a missing thrombospondin type-1 (TSP1) repeat in the deduced *Hc*-NAS-33, compared with the domain architecture of *Ce*-NAS-33 (Figure 1A). The alignment of protein sequence with other nematode homologs showed a relatively high similarity within the predicted domains (Supplementary Figure 2).

### *nas-33* Is Highly Expressed During Late Lethargus

Developmental transcription analyses showed predominant gene expression of *nas-33* in the larval (i.e., L1, L2, L3, and L4) stages of both *C. elegans* and *H. contortus* (Figures 1B,D). Specifically, four transcriptional peaks were identified for *Ce-nas-33* across the development from the activated L1 stage to the adult stage of *C. elegans* (Figure 1B). In particular, *Ce-nas-33* appeared to play a role in the late lethargus phase, with reference to the transcriptions of marker genes (i.e., *nas-37*, *col-12*, and *trxr-1*) for molting process (i.e., apolysis, late lethargus and ecdysis) (Figure 1C). A transcriptional peak was also found during the L1-L2 molting of *H. contortus* (Figure 1E). By contrast, unlike the peaked transcriptional level of *nas-33* between apolysis and late lethargus steps in *C. elegans*, *Hc-nas-33* appeared to be highly transcribed between late lethargus and ecdysis processes in *H. contortus*, which were defined based on the transcriptions of marker genes *nas-37*, *col-12*, and *gsr-1* (Figure 1F).

### Knockdown of *nas-33* Leads to Molting Defects in *H. contortus*

**FIGURE 2:**
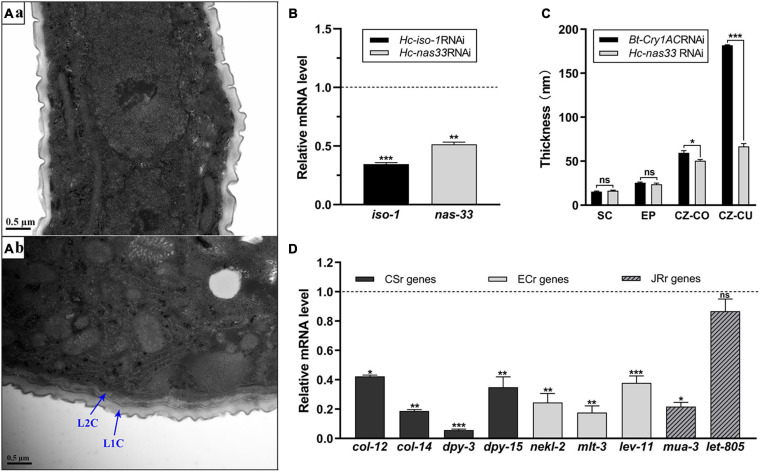
Structural and transcriptional analyses of RNA interference-treated *Haemonchus contortus*. **(A)** Transmission electron microscopy of *Bt-Cry1AC* RNAi-treated (a) and *nas-33* RNAi-treated (b) worms. Scale bar: 0.5 μm. L1C: L1 cuticle; L2C: L2 cuticle. **(B)** Gene knockdown analysis of *iso-1* (positive control) and *nas-33* in RNAi-treated worms, with reference to the transcriptions of these genes in the untreated worms. **(C)** Cuticle thickness (measured by ImageJ) of the second larval stage of *H. contortus* after RNAi. SC, surface coat; EP, epicuticle; CZ-CO, collagen rich layer of cortical zone; CZ-CU, cuticlin rich layer of cortical zone. **(D)** Relative transcriptional changes of molting related genes in RNAi-treated worms, compared with that in untreated worms. CSr genes, cuticle synthesis related genes (*col-12*, *col-14*, *dpy-3*, *dpy-15*); ECr genes, ecdysis related genes (*nekl-2*, *mlt-3*, *lev-11*); JRr genes, junction remodeling related genes (*mua-3*, *let-805*). Student’s *t*-test is used for the statistical analysis between treated and untreated worms. *P < 0.05; **P < 0.01; ***P < 0.001; ns, no significance. Dotted line represents the transcriptional level in negative control worms.

**FIGURE 3:**
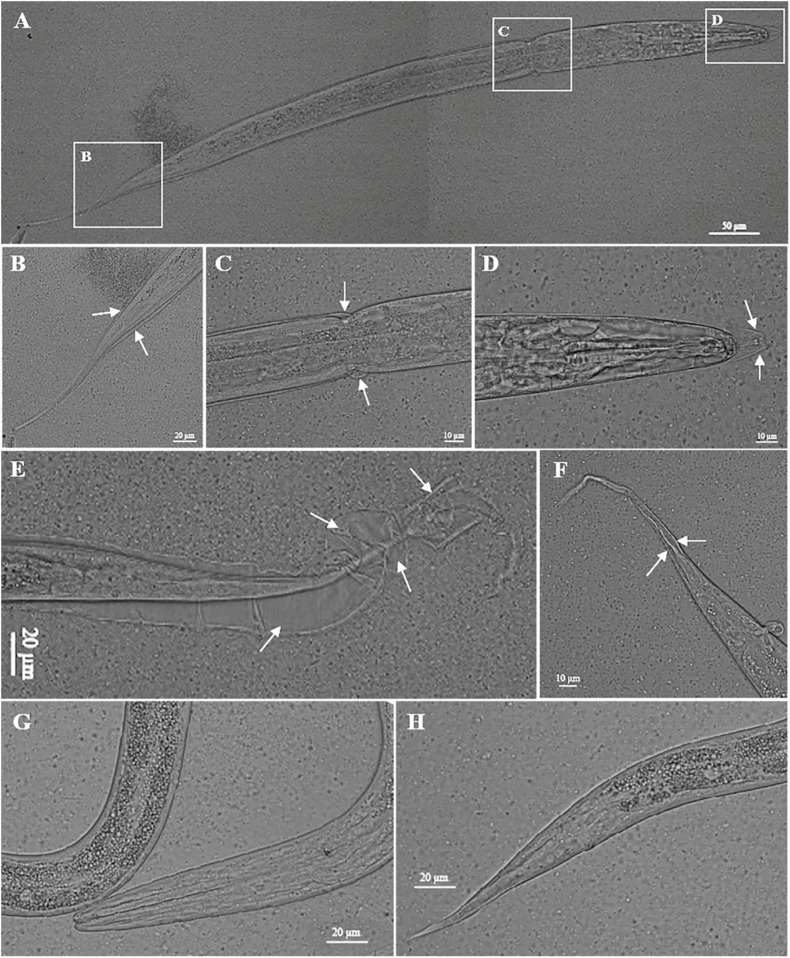
Knockdown of *nas-33* leads to molting defects in *Haemonchus contortus*. **(A–D)** Defects in the first molting of *H. contortus* led to larva death. **(E,F)** Defects in the first molting of *H. contortus* resulted in attachment of the old cuticle to the second stage larva. Cuticles failed to be shed are marked with white arrows. **(G,H)** Phenotype of untreated larvae of *H. contortus*.

Compared with the untreated worms, two layers of cuticles were observed in the *nas-33* RNAi-treated larvae of *H. contortus* (Figure 2A). To confirm the association between phenotypic change and RNAi-mediated knockdown of *nas-33* in *H. contortus*, transcriptional levels of *nas-33* and *iso-1* (positive control) were measured by qRT-PCR. Both of the two genes showed a significant (*P* < 0.05) decrease in the RNAi-treated larvae (Figure 2B), with the thickness of the L2 cuticle significantly (*P* < 0.001) thinner than that of untreated larvae (Figure 2C). In addition, successful gene knockdown of *nas-33* led to marked transcriptional alterations of genes involved in the molting process of *H. contortus*. Specifically, lower transcriptional level of *nas-33* (*P* < 0.01) in the RNAi-treated worms was linked to significant downregulation of four genes *col-12* (*P* < 0.05), *col-14* (*P* < 0.01), *dpy-5* (*P* < 0.001), and *dpy-13* (*P* < 0.01) involved in cuticle synthesis, three genes *nekl-2* (*P* < 0.01), *mlt-3* (*P* < 0.01), and *lev-11* (*P* < 0.001) involved in ecdysis, and one gene *mua-3* (*P* < 0.05) involved in junction remodeling (Figure 2D). In addition, suppression of *nas-33* significantly compromised the molting process of L1s in *H. contortus*. Particularly, gene knockdown of *Hc*-*nas-33* resulted in obvious molting defects (e.g., failure of shedding the old cuticle and corset phenotype), compared with negative control (Figure 3).

### *Hc-NAS-33* Interacts With a G Protein Subunit *in vitro* and *in vivo*

**FIGURE 4:**
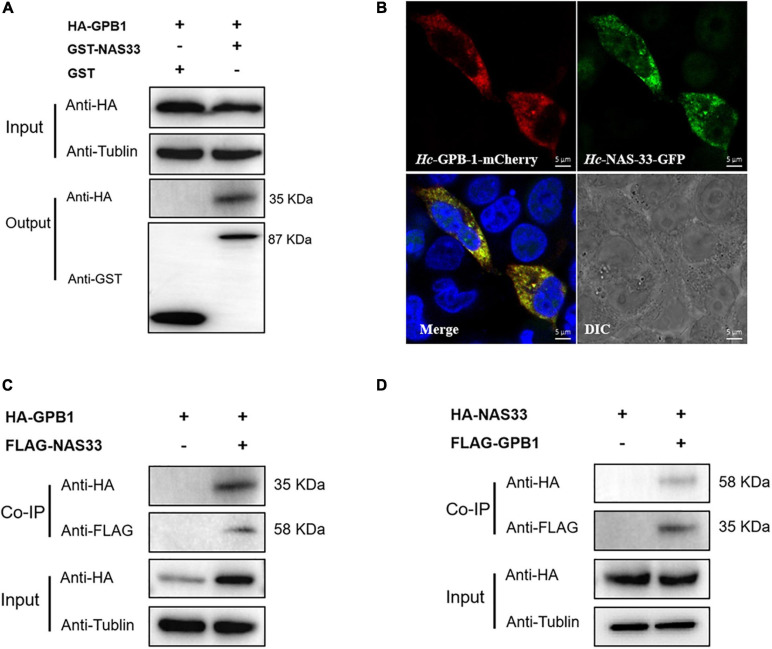
*Hc*-NAS-33 interacts with *Hc*-GPB-1 *in vitro* and *in vivo*. **(A)** Pull-down assay showing the interaction of *Hc*-NAS-33 and *Hc*-GPB-1. **(B)** Co-localization of *Hc*-NAS-33 and *Hc*-GPB-1 in HEK 293T cells. Scale bar: 5 μm. **(C,D)** Co-IP assay verifying the interaction of *Hc*-NAS-33 and *Hc*-GPB-1 *in vivo*.

**FIGURE 5:**
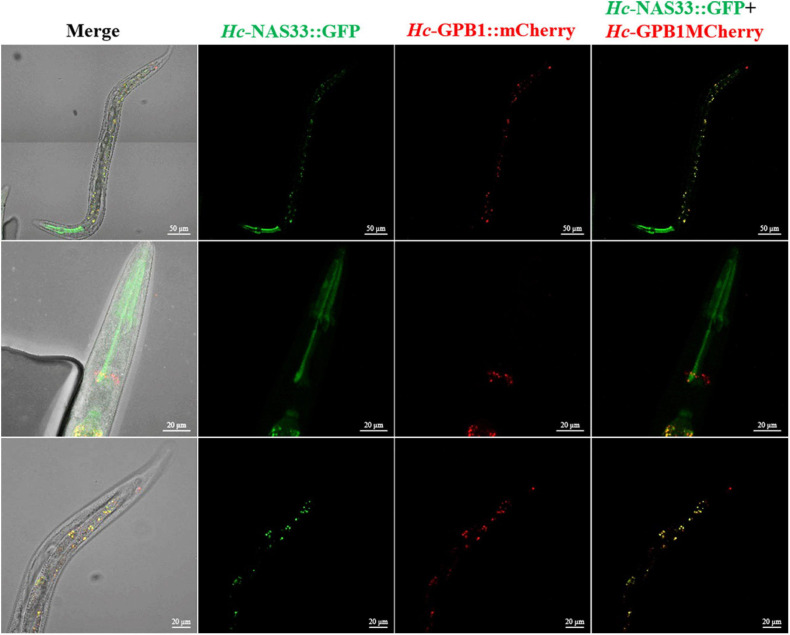
Co-localization of *Hc*-NAS-33 and *Hc*-GPB-1 in *C. elegans*. GFP, green fluorescent protein; mCherry, monmer cherry fluorescent protein.

By screening the yeast two-hybrid cDNA library of *H. contortus*, several proteins were identified as candidates interacting with *Hc-*NAS-33, including a guanine nucleotide-binding protein subunit beta-1 (GPB-1). The interaction between *Hc-*NAS-33 and *Hc-*GPB-1 was verified by the GST pull-down assay *in vitro* (Figure 4A), and confirmed with the co-IP assay *in vivo* (Figure 4). In addition, protein expression analyses of *Hc-*NAS-33 and *Hc*-GPB-1 in both HEK 293T cells and tissues of *C. elegans* to some extent showed a similar protein distribution. In cells, *Hc*-NAS-33 was consistently colocalized with *Hc*-GPB-1 in the cytoplasm (Figure 4B), whereas in worms, scattered co-localization of *Hc*-NAS-33 and *Hc*-GPB-1 was observed in the intestine of adult worms but not in the pharynx area (Figure 5). Low efficiency and no activity were observed for the possible promoter sequences of *Hc-nas-33* and *Hc-gpb-1*. Driven by promoters of *Ce-nas-33* and *Ce*-*gpb-1*, heterologous protein expression of *Hc*-NAS-33 and *Hc*-GPB-1 were achieved in *C. elegans*, and confirmed by western blot (Supplementary Figure 3). Tissue distribution of *Hc*-GPB-1 is mostly consistent with the sites of *Ce*-*gpb-1* promoter activity in worms, whereas interestingly, a discrepancy between *Ce-nas-33* promoter activity and *Hc*-NAS-33 protein distribution was identified (Supplementary Figure 4).

### Knockdown of NAS-33 and GPB-1 Blocked Development and Survival of Infective Larvae

**FIGURE 6:**
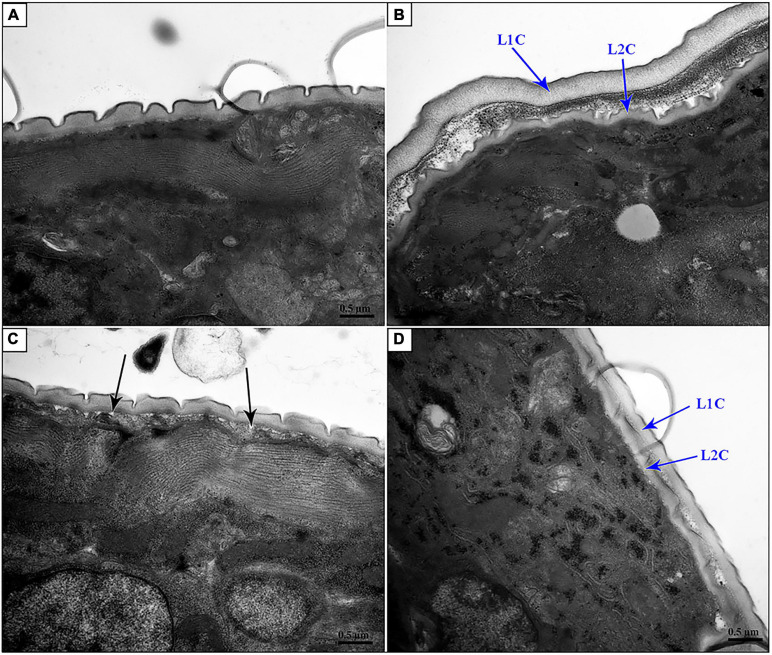
Transmission electron microscopy of *nas-33* and *gpb-1* RNA interference-treated *Haemonchus contortus*. **(A)** Cuticle structure of *Bt-Cry1AC* RNAi-treated worm. **(B)** Two layers of cuticles of *nas-33* RNAi-treated worm. **(C)** Loose connection between cuticle and epidermis in *gpb-1* RNAi-treated worm. **(D)** Two layers of cuticles and loose connection between cuticle and epidermis of *nas-33* and *gpb-1* RNAi-treated worm. L1C, L1 cuticle; L2C, L2 cuticle. Black arrows point to the loose connections between cuticle and epidermis. Scale bar: 0.5 μm.

**FIGURE 7:**
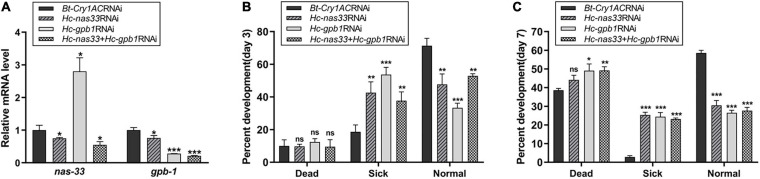
Effects of *nas-33* and *gpb-1* RNA interference on the development and survival of *Haemonchus contortus*. **(A)** Influences of *nas-33* or *gpb-1* RNAi on the relative mRNA levels (normalized by the transcriptional level of *Bt-Cry1AC*) of *nas-33* and *gpb-1*, respectively. **(B)** Influences of separate (*Hc-nas-33* or *Hc-gpb-1*) and simultaneous (*Hc-nas-33* + *Hc-gpb-1*) RNAi on the development and survival of *H. contortus* on day 3. **(C)** Influences of separate (*Hc-nas-33* or *Hc-gpb-1*) and simultaneous (*Hc-nas-33* + *Hc-gpb-1*) RNAi on the development and survival of *H. contortus* on day 7. Sick phenotype includes developmental delay, decreased mobility, abnormality and molting defect. Student’s *t*-test is used for the statistical analysis between treated and negative control. *P < 0.05; **P < 0.01; ***P < 0.001; ns, no significance.

In particular, suppression of *Hc-nas-33* and *Hc-gpb-1* led to obvious molting defects in treated *H. contortus* (Figure 6). Specifically, in *nas-33* RNAi-treated worms, both old and new cuticles were found closely attached to the hypodermis (Figure 6B). In *gpb-1* RNAi-treated worms, only one layer of cuticle was observed, with a loose connection to the hypodermis (Figure 6C). In *nas-33* and *gpb-1* RNAi-treated worms, both two layers of the cuticle and a loose connection to the hypodermis were identified (Figure 6D). In particular, compared with negative control, independent RNAi of *gpb-1* was linked to a significant (*P* < 0.05) upregulation of *nas-33* in treated worms, whereas knockdown of *nas-33* resulted in significant (*P* < 0.05) downregulation of *gpb-1* in *H. contortus*. Notably, simultaneous RNAi of *nas-33* and *gpb-1* significantly enhanced the knockdown efficacies of both *nas-33* (*P* < 0.05) and *gpb-1* (*P* < 0.0001) (Figure 7A).

Defects in larval molting resulted in developmental and survival variations in treated *H. contortus* (Figures 7B,C). In particular, simultaneous knockdown of *nas-33* and *gpb-1* resulted in delayed larval development, decreased mobility and sickness. Compared with negative control, increased number (∼40%) of sick larvae (*P* < 0.01) and decreased healthy larvae (*P* < 0.01) were found in RNAi-treated groups on day 3 (Figure 7B), and increased number (∼50%) of dead larvae (including the free-living L2s and the infective L3s) and decreased number of healthy larvae (*P* < 0.001) in treated groups on day 7 (Figure 7C). No significant difference was found between independent and simultaneous RNAi of *nas-33* and *gpb-1*, in terms of phenotypic changes.

## Discussion

In this study, we report an essential zinc metalloprotease NAS-33 in nematode species. Our findings elucidated that this metalloprotease is likely to play a role in the larval molting process of the free-living *C. elegans* and the parasitic *H. contortus*. In particular, *Hc*-NAS-33 interacts with *Hc*-GPB-1 to control shedding of the old cuticle and remodeling of the connection between the new cuticle and the hypodermis of worms. Suppression of the *Hc*-NAS-33-GPB-1 module resulted in molting defects and a moderate lethal phenotype, suggesting the essentiality of *nas* genes in nematodes.

The astacin-like protein coding gene *nas-33* is relatively conserved in free-living and parasitic nematodes. In *C. elegans*, *nas-35*, *-36*, and -*37* encode astacins of subgroup V (NAS-33 to -38) which have the N-terminal astacin-like, C-terminal EGF (epidermal growth factor), CUB (C1r/C1s, embryonic sea urchin protein Uegf, Bmp-1) and TSP1 domains in order, implying an essential role of astacins of subgroup V in the molting, survival and development of nematodes. Compared with the domain architecture of *Ce*-NAS-33, *Hc*-NAS-33 lacks a TSP1 domain. This domain is usually found in extracellular matrix proteins (Zhang et al., 2020) and a number of proteins involved in the complement pathway (Patthy, 1988). In particular, the thrombospondin type 1 repeat containing proteins ADAMTS (a disintegrin-like and metalloprotease domain) have been proved to be principal mediators of ECM destruction (Apte and Parks, 2015; Liu et al., 2015). In nematodes, cuticle components are synthesized, secreted and modified in the extracellular matrix, and TSP1 of *Ce*-NAS-33 might function in the modification and arrangement of cuticle proteins during late lethargus. However, it is still not clear whether the difference in NAS-33 protein sequence is associated with the unique life cycle and living conditions of parasitic nematodes.

The gene *nas-33* appears to play a role in the molting processes of *H. contortus*. First, predominant transcription of *nas-33* in larval stages indicated that this gene might play roles in larvae development and survival. In *C. elegans*, genes involved in molting (apolysis, late lethargus and ecdysis) usually have a dynamic expression pattern (Hendriks et al., 2014; Turek and Bringmann, 2014). For instance, higher mRNA levels of *Ce-nas-33* were detected in the apolysis and late lethargus stages. However, apart from body-size changes, no significant difference related to molting was observed in *C. elegans* after knockdown of *Ce-nas-33* (Supplementary Figure 5), which might be explained by functional redundancy of *nas* genes in this free-living nematode. Interestingly, it was found that the mRNA level of *Hc-nas-33* was higher in the late lethargus and early ecdysis (based on the transcription of marker genes *nas-37*, *col-12* and *gsr-1*) in *H. contortus*, which is different from that of *Ce-nas-33* in *C. elegans*, indicating subtle functional differences in molting between these two species. Second, downregulation of collagen-associated genes (*col-12*, *col-14*, *dpy-5*, and *dpy-13*; Johnstone and Barry, 1996; Mcmahon et al., 2003; Page and Johnstone, 2007) in RNAi-treated worms suggests that *Hc-nas-33* is required in the synthesis of cuticle structure elements (Cox et al., 1981; Page, 2001), which is further confirmed by the change of cortical zone thickness. In addition, it was reported that *nekl-2* and *mlt-3* genes involved in nekl-mlt kinase network (Yochem et al., 2015; Lazetic and Fay, 2017), and *lev-11* involved in muscle contraction spinning and flipping behavior (Frand et al., 2005; Barnes et al., 2018; Watabe et al., 2018) are essential for molting in *C. elegans*. Reduced expression of *nekl-2*, *mlt-3* and *lev-11* were observed in the *Hc-nas-33*RNAi experiment, indicating that *Hc-nas-33* might be involved in ecdysis via muscle contraction regulation. These findings indicate that *Hc-nas-33* and associated metalloproteases might be a target for reducing the population of *H. contortus* infected larvae, suggesting a possible approach to the prevention of haemonchosis, although it warrants further investigations.

*Hc*-NAS-33 and *Hc*-GPB-1 are required for cuticle synthesis and cuticle-epidermis linkage remodeling. This statement can be supported by the decreased thickness of cortical zone in *Hc-nas-33* RNAi-treated worms and decreased thickness of both epicuticle and cortical zone in *gpb-1* RNAi-treated worms (Supplementary Figure 6A). These results indicate that *Hc-*NAS-33 and *Hc-*GPB-1 are related to protein components synthesis, with *Hc-*GPB-1 likely required for lipid and glycolipid synthesis during the molting process. Additionally, it has been demonstrated that *mua-3* and *let-805/myotactin* are component of hemidesmosome-like structures (HDLSs) through which the epidermis and cuticle are attached to each other. MUA-3 may help to link collagens in the basal zone to the epidermal cytoskeleton (Bercher et al., 2001), and LET-805 may guide the remodeling of basement membrane attachments during molting (Hresko et al., 1999). In the current work, silencing of *Hc-nas-33* led to a predominant downregulation of *mua-3*, whereas knockdown of *Hc-gpb-1* was linked to *let-805* (Supplementary Figure 6B), suggesting their roles in both cuticle synthesis and the remodeling of basement membrane attachments.

Apart from molting, NAS-33 and GPB-1 might play additional roles in nematode species. This is because that the two proteins were partially co-localized in worms. Little has been reported on other functional roles of NAS-33, although it might play a role in embryogenesis or hatching as transcription of this gene was detected in *H. contortus* eggs. By contrast, GPB-1 expression has been observed in the neurons, hypodermal seam cells, gonad and vulva in both larval and adult stages of *C. elegans*. Such expression pattern is consistent with the phenotypes including abnormalities in early embryogenesis, sterility and abnormalities in the germ line caused by GPB-1 depletion (Zwaal et al., 1996). Additionally, the distribution of GPB-1 at the cell membrane is dynamic and asymmetric during the division of one-cell stage *C. elegans* embryos (Thyagarajan et al., 2011). However, these functional roles of NAS-33 or GPB-1 in *H. contortus* and associated parasitic nematodes warrants further investigations.

In conclusion, we identified an essential astacin protein NAS-33 in nematode species. Suppression of this protein and associated G protein subunit resulted in molting defect and death of infective larvae of a highly pathogenic strongylid nematode. Our work provides comprehensive insights into the essentiality of *nas* gene family in nematode molting and survival, and thereby lays a foundation for the discovery of potential targets for the prevention of parasitic diseases of socioeconomic significance.

## Data Availability Statement

The raw data supporting the conclusions of this article will be made available by the authors, without undue reservation.

## Ethics Statement

The animal study was reviewed and approved by the Experimental Animal Ethics Committee, Zhejiang University.

## Author Contributions

YH, XC, and AD designed the research. YH, JW, and DT conducted the experiments. YY, FW, and HZ provided regents. JZ and JW analyzed the data. YH and GM wrote the manuscript. XC, GM, and AD offered advice during the research and provided financial support. All authors read and approved the final manuscript.

## Conflict of Interest

The authors declare that the research was conducted in the absence of any commercial or financial relationships that could be construed as a potential conflict of interest.

## Footnotes

1 https://www.sanger.ac.uk

2 https://www.ncbi.nlm.nih.gov/Structure/cdd/wrpsb.cgi

3 http://www.ebi.ac.uk/interpro/scan.html

## Supplementary Material

Supplementary Figure 1 Isolation of *Hc-nas-33* DNA and cDNA from *Haemonchus contortus.* **(A)** 5′- RACE. **(B)** 3′- RACE. **(C)** 5′- Genome Walking. **(D)** 3′- Genome Walking. **(E)** Cloning of *Hc-nas-33* full length coding sequence by overlapping PCR. 1, 1–213 nucleotide sequence of *Hc-nas-33*; 2, 207–1,569 nucleotide sequence of *Hc-nas-33*; 3, full length coding sequence of *Hc-nas-33.* SP, specific primer; M, DNA marker.

Supplementary Figure 2 Characterization of *Hc-nas-33* in *Haemonchus contortus*. **(A)** Schematic diagram of *Hc-nas-33* gene structure (GenBank accession No. MT891117). Black blocks represent exons and gray blocks represent non-coding 5′- and 3′-untranslated region (UTR) sequence. **(B)** Functional domain prediction and alignment of amino acid sequence of *Hc*-NAS-33 and homologs in other nematode species.

Supplementary Figure 3 Western blotting of *Hc*-NAS-33 and *Hc*-GPB-1 expressed in transgenic worms. M, standard protein marker.

Supplementary Figure 4 Promoter activity analysis of *pCe-nas-33*, *pHc-nas-33*, and *pCe-gpb-1* in *Caenorhabditis elegans*. **(A–C)** Massive expression of *pCe-nas-33*:GFP in the epidermis of *C. elegans*. **(D,E)** Weak expression of *pHc-nas-33*:GFP in several intestine cells. **(F,G)** Specific expression of *pCe-gpb-1*:mCherry in the head and tail of *C. elegans*.

Supplementary Figure 5 Effects of *Ce-nas-33* knockdown on *Caenorhabditis elegans*. **(A)** Development of vulva showed no significant difference after *Ce-nas-33* RNAi compared with negative control (*Bt-Cry1AC*). **(B,C)** Morphological changes (body width and body length) at 36 and 48 h after *Ce-nas-33* RNAi treatment. Student’s *t*-test is used for the statistical analysis between treated and negative control. ^∗∗∗^*P* < 0.001.

Supplementary Figure 6 Analysis of cuticle thickness and junction remodeling related genes after *gpb-1* RNA interference. **(A)** Cuticle thickness (measured by ImageJ) changes of the second stage larva after *gpb-1* RNAi. SC, surface coat; EP, epicuticle; CZ-CO, collagen rich layer of cortical zone; CZ-CU, cuticlin rich layer of cortical zone. **(B)** Influences of *gpb-1* RNAi on the transcription of junction remodeling related genes (*mua-3* and *let-805*). Student’s *t*-test is used for the statistical analysis between treated and negative control. ^∗∗^*P* < 0.01; ^∗∗∗^*P* < 0.001; ns, no significance.

Supplementary Table 1 Information on primer sets used in this study.
