# Supplementary figures and images for: A Zinc Metalloprotease nas-33 Is Required for Molting and Survival in Parasitic Nematode Haemonchus contortus
Source: Front Cell Dev Biol. 2021 Jul 13;9:695003. doi: 10.3389/fcell.2021.695003 (PMC8313830; doi:10.3389/fcell.2021.695003)

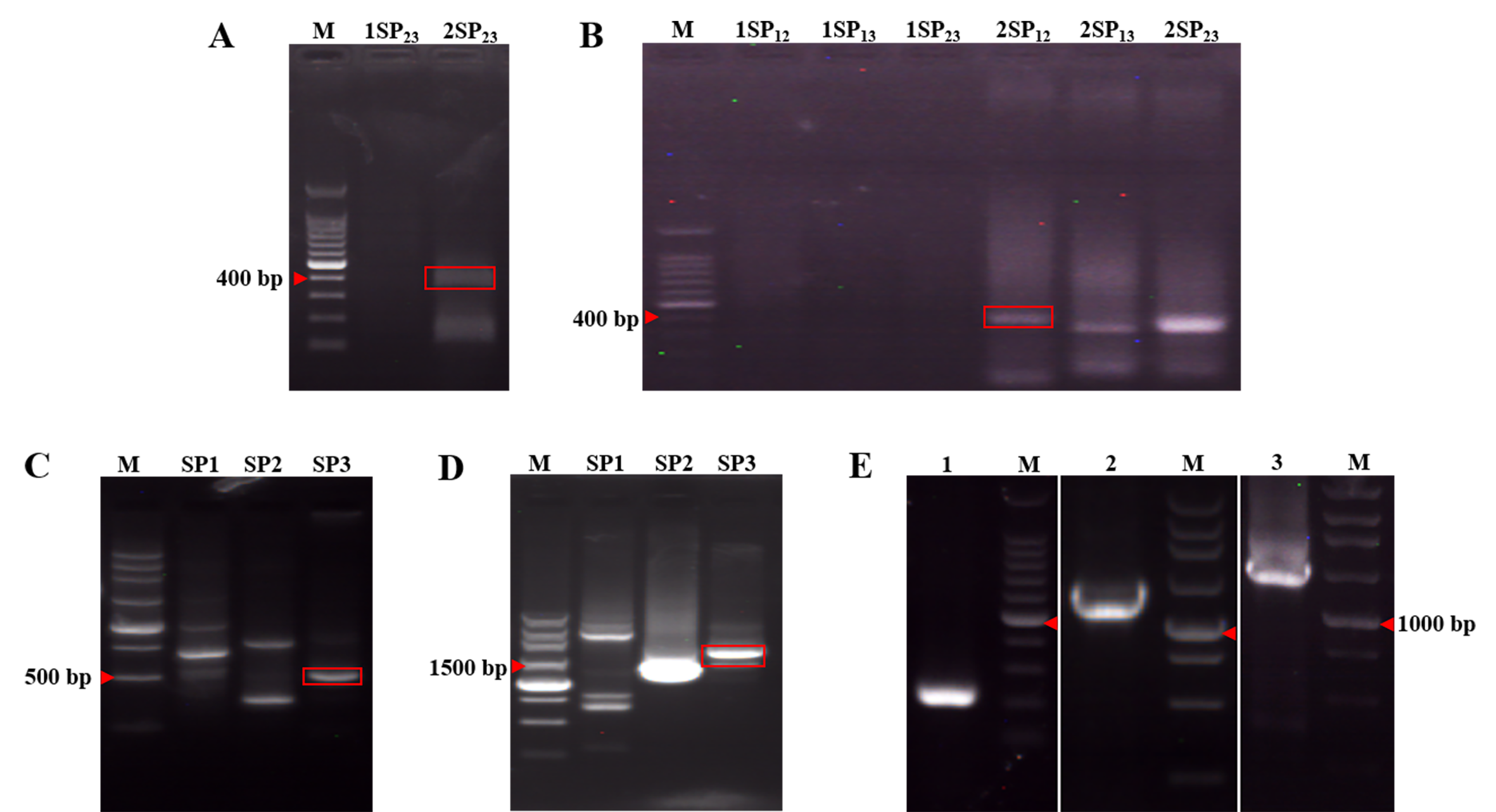

Supplement: Supplementary Figure 1 — Isolation of Hc-nas-33 DNA and cDNA from Haemonchus contortus. (A) 5′- RACE. (B) 3′- RACE. (C) 5′- Genome Walking. (D) 3′- Genome Walking. (E) Cloning of Hc-nas-33 full length coding sequence by overlapping PCR. 1, 1–213 nucleotide sequence of Hc-nas-33; 2, 207–1,569 nucleotide sequence of Hc-nas-33; 3, full length coding sequence of Hc-nas-33. SP, specific primer; M, DNA marker. [file Image_1.TIF]

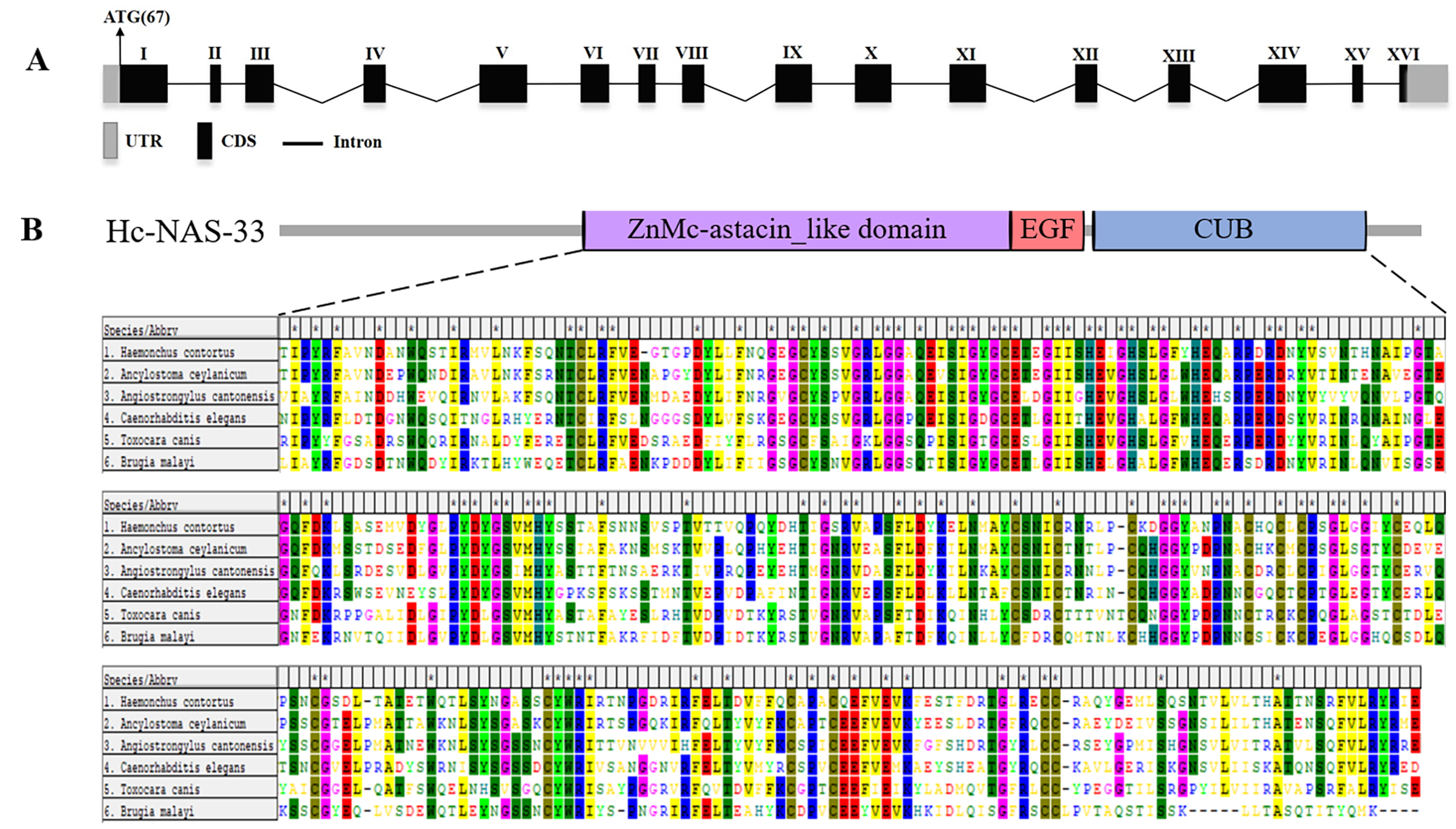

Supplement: Supplementary Figure 2 — Characterization of Hc-nas-33 in Haemonchus contortus. (A) Schematic diagram of Hc-nas-33 gene structure (GenBank accession No. MT891117). Black blocks represent exons and gray blocks represent non-coding 5′- and 3′-untranslated region (UTR) sequence. (B) Functional domain prediction and alignment of amino acid sequence of Hc-NAS-33 and homologs in other nematode species. [file Image_2.TIF]

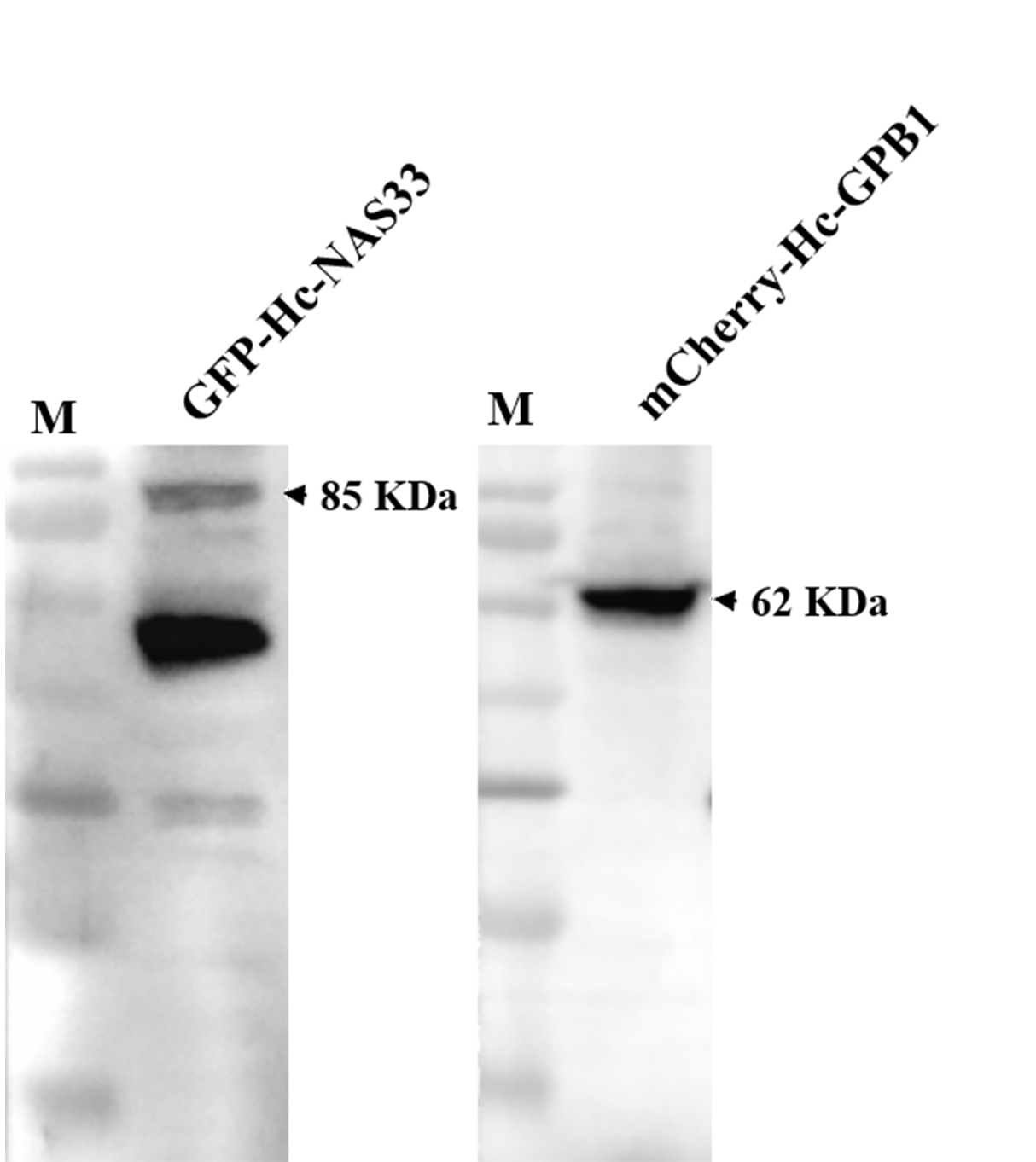

Supplement: Supplementary Figure 3 — Western blotting of Hc-NAS-33 and Hc-GPB-1 expressed in transgenic worms. M, standard protein marker. [file Image_3.TIF]

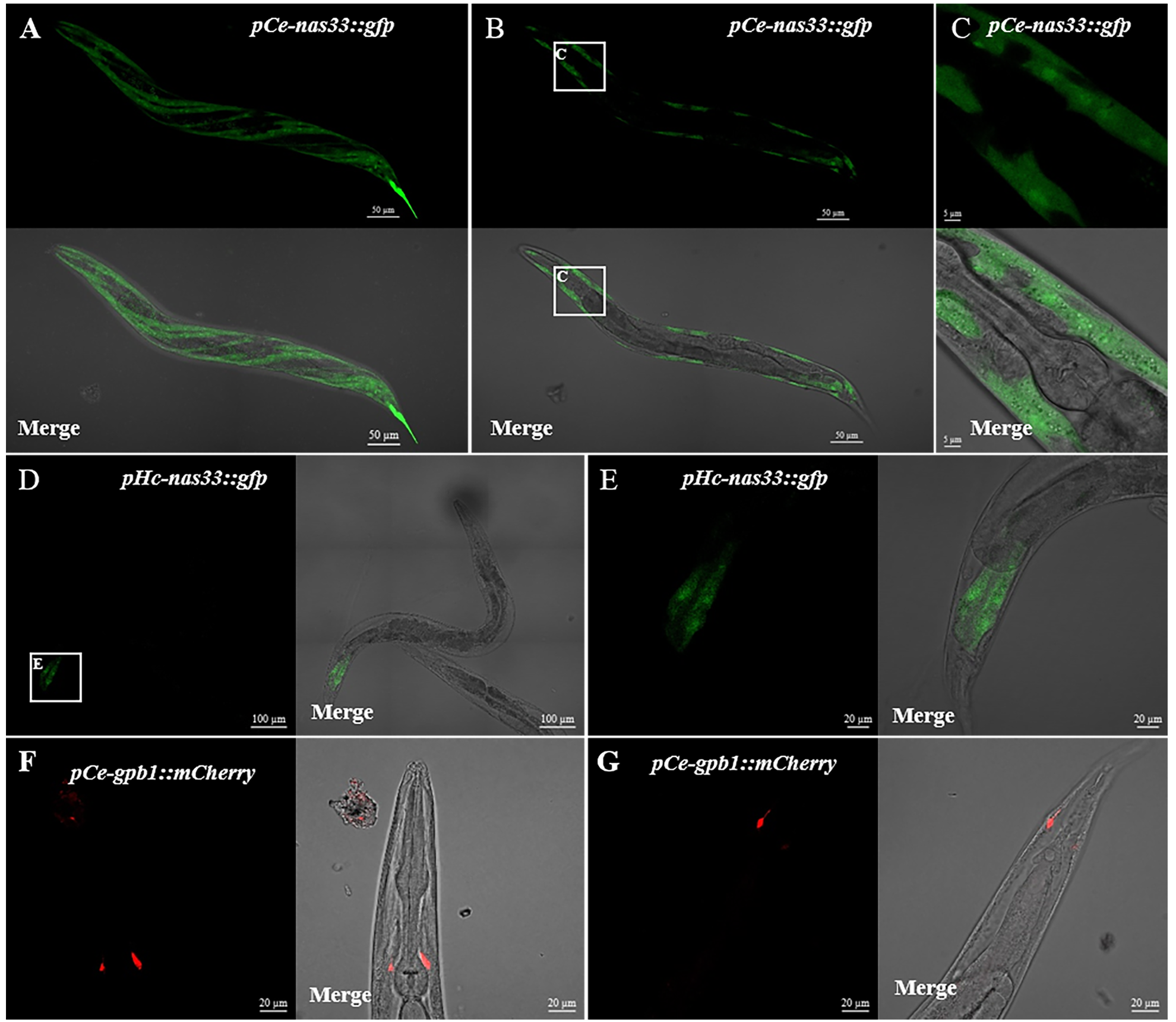

Supplement: Supplementary Figure 4 — Promoter activity analysis of pCe-nas-33, pHc-nas-33, and pCe-gpb-1 in Caenorhabditis elegans. (A–C) Massive expression of pCe-nas-33:GFP in the epidermis of C. elegans. (D,E) Weak expression of pHc-nas-33:GFP in several intestine cells. (F,G) Specific expression of pCe-gpb-1:mCherry in the head and tail of C. elegans. [file Image_4.TIF]

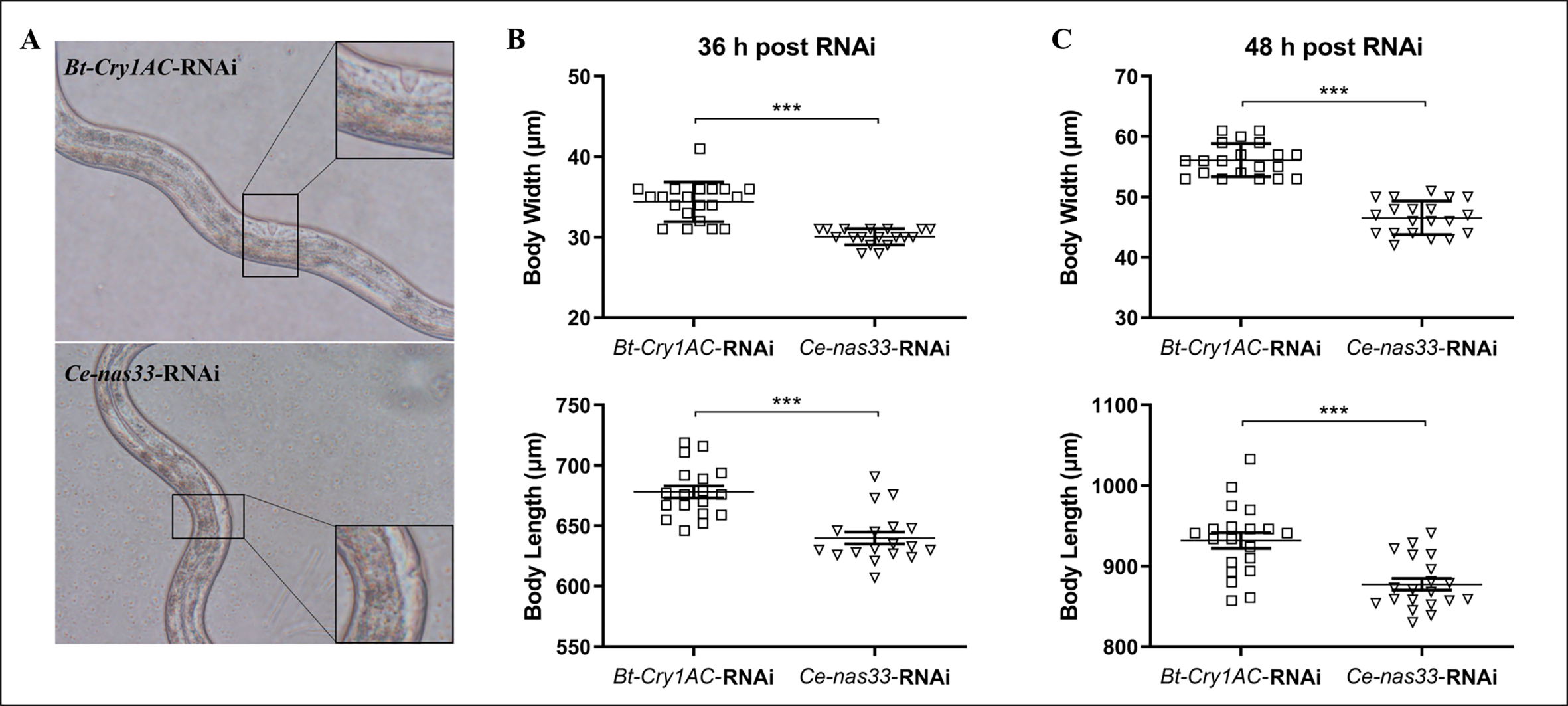

Supplement: Supplementary Figure 5 — Effects of Ce-nas-33 knockdown on Caenorhabditis elegans. (A) Development of vulva showed no significant difference after Ce-nas-33 RNAi compared with negative control (Bt-Cry1AC). (B,C) Morphological changes (body width and body length) at 36 and 48 h after Ce-nas-33 RNAi treatment. Student’s t-test is used for the statistical analysis between treated and negative control. ∗∗∗P < 0.001. [file Image_5.TIF]

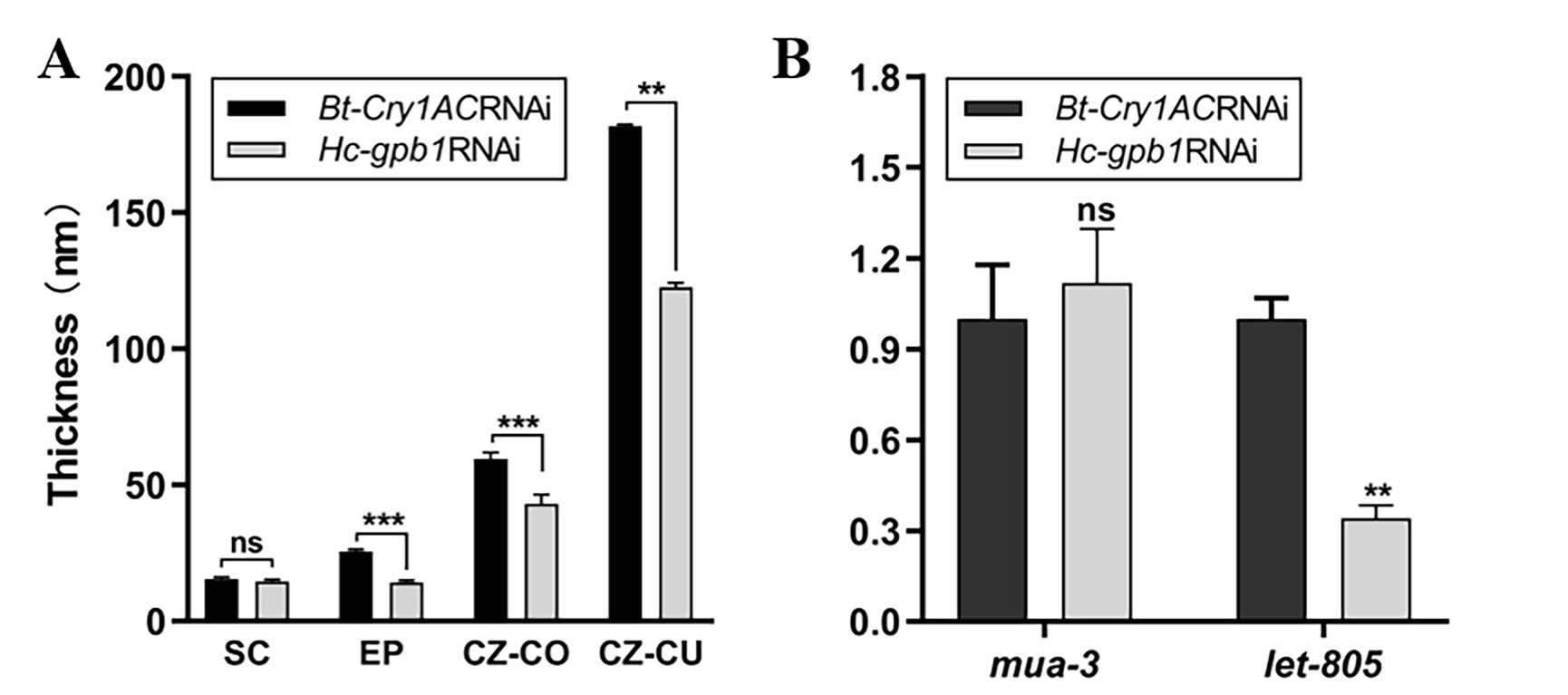

Supplement: Supplementary Figure 6 — Analysis of cuticle thickness and junction remodeling related genes after gpb-1 RNA interference. (A) Cuticle thickness (measured by ImageJ) changes of the second stage larva after gpb-1 RNAi. SC, surface coat; EP, epicuticle; CZ-CO, collagen rich layer of cortical zone; CZ-CU, cuticlin rich layer of cortical zone. (B) Influences of gpb-1 RNAi on the transcription of junction remodeling related genes (mua-3 and let-805). Student’s t-test is used for the statistical analysis between treated and negative control. ∗∗P < 0.01; ∗∗∗P < 0.001; ns, no significance. [file Image_6.TIF]
